# Bellerophon: a hybrid method for detecting interchromo-somal rearrangements at base pair resolution using next-generation sequencing data

**DOI:** 10.1186/1471-2105-14-S5-S6

**Published:** 2013-04-10

**Authors:** Matthew Hayes, Jing Li

**Affiliations:** 1 Department of Electrical Engineering and Computer Science, Case Western Reserve University, 10900 Euclid Ave., Cleveland, OH, USA

## Abstract

**Background:**

Somatically-acquired translocations may serve as important markers for assessing the cause and nature of diseases like cancer. Algorithms to locate translocations may use next-generation sequencing (NGS) platform data. However, paired-end strategies do not accurately predict precise translocation breakpoints, and "split-read" methods may lose sensitivity if a translocation boundary is not captured by many sequenced reads. To address these challenges, we have developed "Bellerophon", a method that uses discordant read pairs to identify potential translocations, and subsequently uses "soft-clipped" reads to predict the location of the precise breakpoints. Furthermore, for each chimeric breakpoint, our method attempts to classify it as a participant in an unbalanced translocation, balanced translocation, or interchromosomal insertion.

**Results:**

We compared Bellerophon to four previously published algorithms for detecting structural variation (SV). Using two simulated datasets and two prostate cancer datasets, Bellerophon had overall better performance than the other methods. Furthermore, our method accurately predicted the presence of the interchromosomal insertions placed in our simulated dataset, which is an ability that the other SV prediction programs lack.

**Conclusions:**

The combined use of paired reads and soft-clipped reads allows Bellerophon to detect interchromosomal breakpoints with high sensitivity, while also mitigating losses in specificity. This trend is seen across all datasets examined. Because it does not perform assembly on soft-clipped subreads, Bellerophon may be limited in experiments where sequence read lengths are short.

**Availability:**

The program can be downloaded from http://cbc.case.edu/Bellerophon

## Background

Genomic structural variants (SV) are widespread, and along with single nucleotide polymorphisms (SNPs), are believed to contribute to phenotypic variation within populations. Many structural variants are inherited. However, somatically-acquired variants could play an important role in the onset and progression of diseases such as cancer [1,2]. Among the classes of structural variants are interchromosomal translocations, which are caused by the fusion of two non-homologous chromosomes. A translocation can disrupt gene function (e.g. a tumor suppressing gene), or it could create a fusion gene that codes for a protein with deleterious functions [3,4]. An example of such a fusion is the Philadelphia chromosome, which is highly associated with chronic myelogenous leukemia [5]. The presence of a somatically-acquired translocation could indicate susceptibility to a particular type of cancer, or it could indicate that the disease has progressed to a certain point [6]. Translocations can thus serve as important clinical markers for determining the cause and nature of certain cancers. It is therefore important to develop efficient methods for locating these variants.

Next-generation sequencing (NGS) allows for the parallel sequencing of entire genomes faster and cheaper than traditional methods for sequencing [7,8]. This has led to the development of several algorithms for detecting structural variation using NGS platforms. Among the methods used for SV detection are BreakdancerMax [9], GASV [10], SVDetect [11], and CREST [12]. SVDetect divides the genome into overlapping windows and predicts structural variants by assessing windows that are linked by anomalously mapped paired reads. BreakdancerMax identifies potential variants by locating regions that contain more abnormally mapped read pairs than is expected. It then uses a Poisson model to calculate a confidence score for each candidate variant. GASV presents a geometric approach to SV detection. This algorithm identifies regions of breakpoint uncertainty and constructs polygons representing these regions. It then finds structural variants by computing the number of intersecting polygons for a given region. The CREST algorithm differs from the previous methods because it does not use abnormally mapped read pairs to find structural variants. Instead, it only uses single reads that contain soft-clipped alignments. Soft-clipped reads contain a contiguous match to the reference, but another contiguous part of the read may be mappable elsewhere. CREST uses these soft-clipped reads to find putative variant breakpoints, and it is thus more effective at finding precise SV boundaries than paired-end approaches.

The aforementioned algorithms can detect several types of structural variants. However, like many paired-end strategies, BreakdancerMax, GASV, and SVDetect cannot determine the precise boundaries of structural variants. Identifying the precise location of variant boundaries is of clinical importance, as the boundaries could provide therapeutic targets in a medical setting. The CREST algorithm can identify precise breakpoints, but since it only depends on the presence of soft-clipped reads to find variants, it could lose sensitivity if SV breakpoints are spanned by only a few soft-clipped reads. To address these issues, we have developed Bellerophon, which predicts interchromosomal translocations by combining paired-end SV detection with breakpoint resolution using clipped alignments. The use of combined SV signatures has been demonstrated by two very recent studies [13,14]. The RetroSeq algorithm uses the combined approach to find genomic transposon insertions, while DELLY predicts deletions, translocations, inversions, and tandem repeats. DELLY was developed independently of Bellerophon, and although we did not compare the two methods directly, DELLY cannot predict interchromosomal insertions.

In addition to breakpoint prediction, our method can also predict the precise nature of a chimeric breakpoint; it attempts to classify each prediction as a 1) balanced translocation, 2) unbalanced translocation, or an 3) interchromosomal insertion. Using simulated datasets and two prostate cancer datasets, we compared Bellerophon to the four aforementioned strategies to assess its ability to predict and locate precise translocation breakpoints. We also created in our simulated data two interchromosomal insertions (where one chromosome "donates" a contiguous segment of genetic material to another chromosome). For both the real and simulated datasets, the alignment results were under-sampled at different rates to assess the performance of each method on varying levels of coverage. For both datasets, Bellerophon had better sensitivity than CREST and similar breakpoint prediction accuracy. Compared to the three paired-end methods, Bellerophon had better breakpoint prediction accuracy and better specificity on the cancer datasets, while having similar sensitivity and specificity on the cancer and simulated datasets respectively.

## Methods

Bellerophon takes as input an alignment file in SAM format [15] generated by a short read aligner. Several algorithms are equipped to align a collection of short sequence fragments to a reference genome. In our experiments, we used the BWA program [16]. When provided a SAM file that is sorted by chromosome and genomic coordinates, Bellerophon then proceeds to the clustering phase.

### Clustering phase

#### *Necessary criteria for a candidate variant*

To identify a candidate interchromosomal variant, Bellerophon first looks for clusters of chimeric read pairs, which are read pairs whose mates are aligned to different chromosomes. Consider a translocation between chromosomes *i *and *j*. The pairs that form the cluster supporting the event must satisfy the following criteria:

1. There must be a collection of reads *R*(*i*) that map to chromosome *i*.

2. There must be a collection of reads *R*(*j*) that map to chromosome *j*, where the reads of *R*(*j*) are the mates of the reads in *R*(*i*).

3. All reads in *R*(*i*) must be mapped closely together and to the same strand.

4. All reads in *R*(*j*) must be mapped closely together and to the same strand.

**Figure 1:**
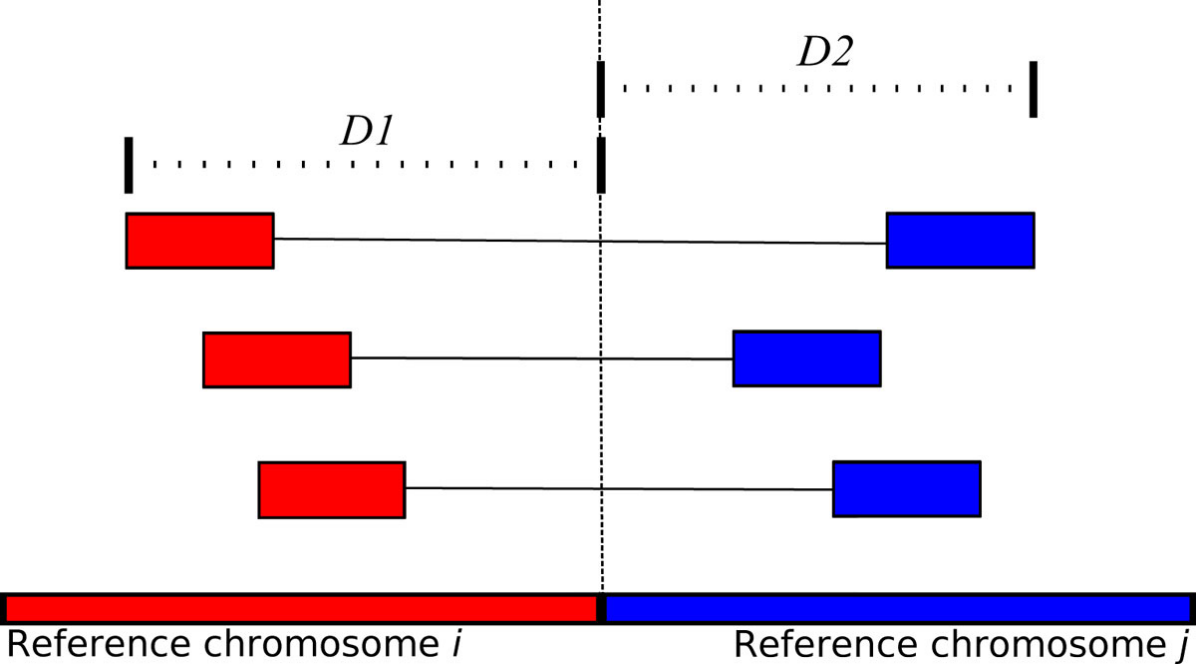
**Translocation captured by a cluster of three chimeric read pairs**. The first set of reads map to the forward strand of chromosome *i*, and the second set map to reverse strand of chromosome *j*. The distance between the outermost reads to the breakpoint are *D1 *and *D2*, for chromosomes *i *and *j *respectively. These distances must be less than or equal to mean + k*stdev.

Criteria 1 and 2 are straightforward because a true variant results in the fusion of two nonhomologous chromosomes. Mate pairs in the cluster must span the chimeric breakpoint, which results in a group of reads mapping to chromosome *i*, and their mates mapping to chromosome *j*. To understand criterion 3, consider the first chimeric pair that spans a particular breakpoint. Let's call this pair *p*. When *p *is mapped to the reference, there is no mapped distance information between its reads since they map to different chromosomes. After mapping, however, we expect that the distance between the first read of *p *and the translocation breakpoint will be within *L *base pairs, where *L *= mean + k*stdev, and *mean *is the average separation distance between mapped read pairs in the dataset, *stdev *is the standard deviation of mapped distances, and *k *is some constant, which for Bellerophon is a user defined value. Figure 1 illustrates a chimeric cluster that implies a true translocation. Since the first encountered read in the cluster is within *L *base pairs of the breakpoint, then subsequent chimeric read pairs must also map within *L *base pairs of the breakpoint. It follows from this observation that all reads in *R*(*i*) will map within *L *base pairs of each other. This also holds the for the reads in *R*(*j*). Furthermore, all the reads in a set must map to a common strand, because if the cluster implies a true translocation, then one of the three scenarios must have occurred.

1. p-arm to q-arm fusion

2. p-arm to p-arm fusion

3. q-arm to q-arm fusion

**Figure 2:**
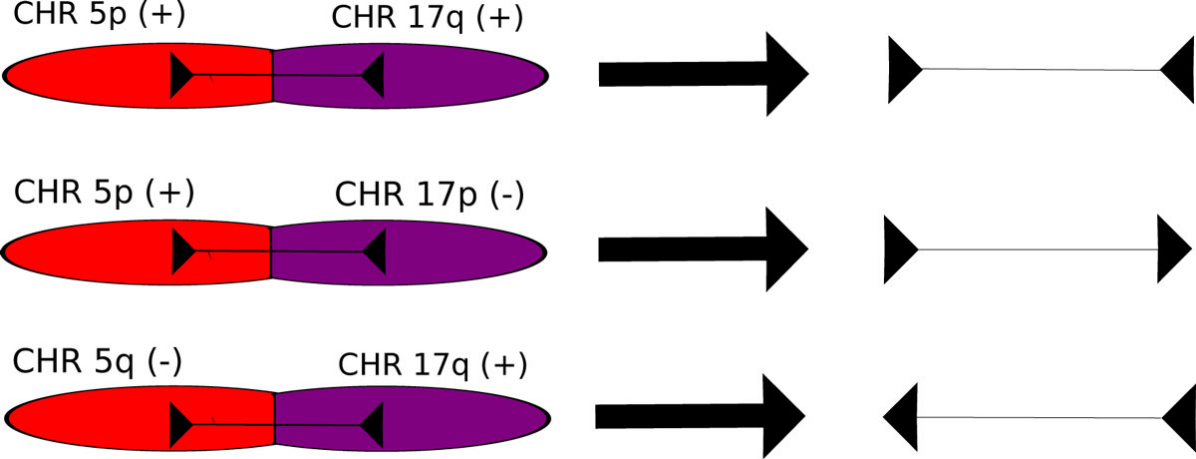
**The three types of translocations and the mapping orientations that result when a pair spans the breakpoint**. These orientations assume that Illumina technologies were used in sequencing. Bellerophon deduces the type of fusion based on the mapping orientation of the pairs in a cluster.

The mapping orientation of the read pairs depends on which chromosomal arms formed the translocation. Figure 2 illustrates this. Lastly, there is criterion 4, but the requirements are the same as that of criterion 3.

#### *The clustering algorithm*

To predict likely translocations, Bellerophon must first find the clusters of chimeric read pairs. It does so by finding collections of chimeric reads that satisfy the four previously mentioned criteria. Essentially, the algorithm collects all chimeric read pairs that map closely together on both ends. The pairs must also share the same two participating chromosomes. If such a collection of read pairs is found, then it possibly denotes a true chromosomal fusion. After a cluster is found by the algorithm, it must then determine which chromosomal arms create the fusion. It does so by examining the orientation of the aligned reads in a cluster, as shown in Figure 2.

#### *Breakpoint resolution*

After finding clusters that could imply a translocation, Bellerophon performs its breakpoint resolution step. In this step, the program will attempt to find the **precise **location on the genome where the chromosomal fusion occurred. This is an improvement over methods that only used paired-end reads, as such methods cannot accurately predict the true breakpoints.

To understand this step of the program, consider a cluster of chimeric pairs *K *which was produced by the clustering algorithm referenced in the previous section, and which has participating chromosomes *i *and *j*. Because of criteria 3 and 4 of the clustering step, we expect that all chromosome *i *reads of *K *will map to within *L *base pairs of the true chromosome *i *breakpoint. Similarly, the chromosome *j *reads will map to within *L *base pairs of the true chromosome *j *breakpoint.

**Figure 3:**
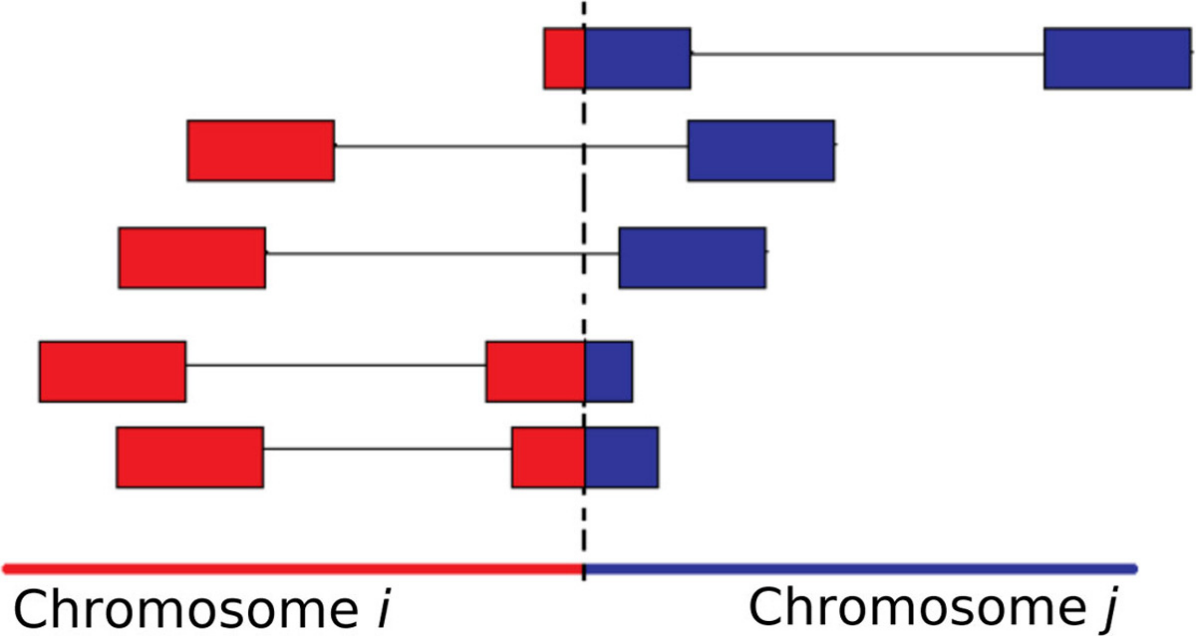
**The formation of soft-clipped reads**. Soft-clipped reads span the translocation boundary between chromosomes *i *and *j*. As a result, these reads may align partially to chromosome *i *and partially to chromosome *j*.

Mapping the breakpoints works as follows: let *clip*(*x,y*) be a soft clipped read where the aligned portion maps to chromosome *x*, and the clipped portion maps to chromosome *y*. Assuming the method is analyzing chromosome *i *alignments, then once the clusters are formed, Bellerophon extends a window *W *from the outermost read in *R*(*i*) to the direction nearest the breakpoint. The size of this window is *L*, and the direction of the window extension depends on the type of chromosome fusion that the method is searching for. The method then performs the same step for chromosome *j*; it extends a window *X *from the outermost read in *R*(*j*) to the direction nearest the breakpoint. The size of *X *is also *L*. Within windows *W *and *X*, Bellerophon then searches for soft-clipped reads, because if there exists a true chimeric breakpoint *b*, then there should be reads at *b *that partially align to both chromosomes *i *and *j*. Specifically, we should have a collection of both *clip*(*i,j *) and *clip*(*j,i*) reads at *b*. Figure 3 illustrates how soft-clipped reads are formed, and how they align to both sides of the variant boundary.

Now that the program has the clipped reads, it continues to the realignment step. The mapped location of the clipped portion of the read is unknown. In other words, once the breakpoint is encountered from both sides, we have *clip*(*i,x*) and *clip*(*j,x*) reads, where *x *is an unknown chromosome. In this step, the algorithm determines the precise location of the clipped subreads so that it can determine *x *and the read's coordinates too. For each soft-clipped read within windows *W *and *X*, if the size of the clipped portion is at least 20 base pairs, then this portion is aligned to the human reference genome using BLAT. We required a threshold of 20 bp because BLAT is best suited to align sequences that are at least 20 base pairs in length [17].

In this step, we attempt to find the precise translocation breakpoints on both sides of the fusion. On the chromosome *i *side of the boundary, the aligned portion of the clipped reads obviously map to chromosome *i*, but the clipped portion of the read should map to chromosome *j*. Similarly for the chromosome *j *side of the boundary, the clipped subreads will map to chromosome *i*, whereas the aligned part of the read will map to chromosome *j*. As a result, we have the following four sets of subreads: *Align*(*i*), *Align*(*j*), *Clipped*(*i*), *Clipped*(*j*). The *Align*(*i*) set contains the subreads whose alignment is to chromosome *i. Align*(*j*) set is similarly defined for chromosome *j*. The *Clipped*(*i*) and *Clipped*(*j *) sets contain the clipped subreads that align to chromosomes *i *and *j*, respectively.

To predict the breakpoint location for chromosome *i*, we calculate the mode of the aligned coordinates of reads in the set *S *= {*Clipped*(*i*) ∪ *Align*(*i*)}. The subreads in *S *may not necessarily align to the same location (due to small indels or mutations), so we assume that the true breakpoint will be the coordinate with the most subreads aligned to it. This step is also performed on the set *T *= {*Clipped*(*j*) ∪ *Align*(*j*)} to predict the precise breakpoint on the chromosome *j *side of the variant.

We use clipped alignment information from both sides of the breakpoint because it is possible that a true breakpoint will be without a sufficient number of clipped reads on either side of the boundary. By using clipped reads from each side, we increase the chances that it is accurately captured from both sides. To call a candidate variant as a predicted interchromosomal variant, there must exist at least one soft-clipped read that realigns to the window *W*, or at least one soft-clipped read that realigns to window *X*. Given the large size of the genome, the probability that even a single clipped sub-read remaps to the windows *X *and *W *is presumably small if no structural variation has occurred. A flowchart of the prediction algorithm is provided in Additional file 1, figure S1.

#### *Chimeric breakpoint classification*

It may be desirable to not only **find **chromosomal fusion boundaries, but to also determine the precise nature of the fusion; does it imply a balanced translocation, unbalanced translocation, or interchromosomal insertion? Most methods for finding chimeric breakpoints do not attempt to answer this question; they are focused on *finding *the boundaries instead of resolving their exact nature (Additional file 1 figure S4). In some experiments, a researcher may want to determine exactly how a chimeric fusion occurred. If it is caused by an interchromosomal insertion (Additional file 1, figure S5), then a chromosome *i *donated a contiguous segment to a non-homologous chromosome *j*, and two chimeric breakpoints are formed from this transfer of material. It is also possible that during the exchange, the orientation of the inserted segment might be inverted with respect to the centromere. This is known as an *inverted insertion*, whereas in the case of no inversion, it is a *direct insertion *[18]. Bellerophon accounts for both cases. The classification algorithm is provided in the Figures S2 and S3. Since interchromosomal insertions can also create fusion genes [19], it may be useful to predict their existence for some experiments.

## Results and discussion

### Variant prediction on two simulated datasets

In our first experiment, we used two simulated datasets to test our program's ability to 1) detect translocation breakpoints and to 2) accurately predict the location of the translocation breakpoints. For the first dataset, we created a simulated test genome by inserting into the human reference genome the variants listed in Table 1. Since balanced translocations are not always entirely reciprocal, we added a 1000 bp duplication to the p-arm of chromosome 6. Duplications and deletions at reciprocal translocation breakpoints occur in some cancers [20], so Bellerophon allows for reciprocal translocation breakpoints to overlap by at most 1 Mb, or to be separated by at most 1 Mb (by default). For the second dataset, we inserted into the reference genome the variants listed in Table 2.

**Table 1:** Structural variants inserted into the first simulated dataset

Chr1	Bkpt1	Strand1	Chr2	Bkpt2	Strand2	Type
9	73,000,000	+	11	63,000,000	+	U

5	40,000,000	+	2	140,000,000	+	U

7	11,000,000	+	12	45,000,000	-	U

10	5,000,000	-	20	15,000,000	+	U

16	6,000,000	-	18	12,000,000	+	U

4	9,000,000	+	17	17,000,000	+	U

3	35,000,000	+	6	14,000,000	+	B

6	14,001,000	+	3	35,000,001	+	B

13	45,000,000	+	**14**	**30,000,000**	+	II

**14**	**30,200,000**	+	13	45,000,001	+	II

**1**	**105,000,000**	-	22	25,000,000	+	II

22	25,000,001	+	**1**	**105,600,000**	-	II

Structural variants inserted into the simulated dataset. U = unbalanced translocation, II = interchromosomal insertion, and B = balanced translocation. For the "II" and "B" variants, the partner breakpoints are listed consecutively. For "II" variants, the donor chromosome and its breakpoint are bolded. Note that the chr3 and chr6 balanced translocation contains a 1000-bp duplication, so it is not entirely reciprocal.

**Table 2:** Structural variants inserted into the second simulated dataset

Chr1	Bkpt1	Strand1	Chr2	Bkpt2	Strand2	Type
15	41,000,000	+	18	50,000,000	+	U

13	31,000,000	+	20	43,000,000	+	U

9	21,000,000	-	17	60,000,000	+	U

21	30,000,000	+	2	35,000,000	-	U

11	11,000,000	+	12	67,000,000	+	U

16	23,000,000	+	7	44,000,001	+	B

7	44,000,000	+	16	23,000,001	+	B

6	92,000,000	-	10	65,000,000	+	U

19	35,000,000	+	14	55,000,000	-	U

For the first dataset, we created simulated paired-end sequence reads from this synthetic data using wgsim. We generated the dataset with 40X sequence read coverage and 100 base pair (bp) reads. It also had a 400 bp average insert size with a standard deviation of 80. The mutation rate was set at 0.001, and among the mutations, the fraction of indels was set to 0.15. The second dataset used the same experimental setup, except for this dataset, we used 75 bp read lengths.

After creating the sequence reads, we aligned them to the human reference genome (NCBI 36) using BWA. After acquiring the alignment results, we created four more datasets by randomly "down sampling" the original alignments at the following rates: 75%, 50%, 25%, and 10%. Each rate is the probability that an aligned mate pair (or single anchoring read) from the original SAM file would be included in the new sampled SAM file. Thus, the resulting files had average coverage of approximately 30X, 20X, 10X, and 4X. After this step, the five datasets were analyzed with the following programs: Bellerophon, GASV, Breakdancer, SVDetect, and CREST. We did not use GASVPro since it is not equipped to handle translocations. On these datasets, we compared each program's ability to predict breakpoints by measuring sensitivity, specificity, and average breakpoint error. For a given SV prediction, the breakpoint error is defined as the difference in base pairs between the true variant boundary and the predicted variant boundary. After identifying the individual breakpoints, we applied the classification algorithm to the prediction results from the 40X coverage alignments.

### Performance on two primary prostate cancer datasets

In our second experiment, we investigated Bellerophon's ability to predict translocations in two prostate cancer datasets PR-0508 and PR-1783 (Berger et al., 2011). These datasets were sequenced using the Illumina GA II sequencer at 30X haploid coverage. The insert sizes were approximately 400 bp and the read lengths were 101 bp. After removing replicate artifacts using the Picard suite, we aligned the sequence reads to the human reference genome. We then applied the same experimental design to the cancer data that was applied to the simulated data described in the previous section. Thus, the resulting sampled alignment files had read depth of approximately 22.5X, 15X, 7.5X, and 3X for the 75%, 50%, 25%, and 10% sampling rates respectively. The list of interchromosomal breakpoints for each dataset is provided in the original study [21].

### Results on simulated data

The results of each method on the simulated datasets are provided in Tables 3 and 4. For the simulated data, all of the methods performed well with regards to specificity. However, Bellerophon had the highest total sensitivity across all datasets. As expected, CREST did not perform well on the 10% dataset. For this low-coverage dataset, the variant breakpoints were spanned by few individual reads. Because CREST only relies on the presence of soft clipped reads, it is susceptible to losses in sensitivity in such data, especially since it requires several soft clipped reads to trigger the assembly portion of its algorithm. Because Bellerophon uses paired reads in addition to soft-clipped reads, it does not require that many individual reads span a variant boundary. For Bellerophon, we required at least one soft-clipped read from either side of the breakpoint and at most five soft-clipped reads from both sides of the breakpoint. This second requirement is for efficiency.

**Table 3:** Simulated dataset 1 results (100 bp reads)

	40X			30X			20X			10X			4X		
**Method**	**SE**	**SP**	**ABE**	**SE**	**SP**	**ABE**	**SE**	**SP**	**ABE**	**SE**	**SP**	**ABE**	**SE**	**SP**	**ABE**

B-phon	12/12	12/12	0.96	12/12	12/12	1.0	12/12	12/12	0.96	11/12	11/11	1.36	5/12	5/5	0.5

SVD	11/12	11/11	2.7-417	11/12	11/11	4.8-405	11/12	11/11	7.4-389	11/12	11/11	14.7-370	8/12	8/9	29-336

BD	12/12	16/16	185.3	12/12	16/16	189.1	11/12	14/14	171.3	10/12	15/15	137.6	4/12	4/4	153

GASV	12/12	12/12	100-226	12/12	12/12	103-245	12/12	12/12	106-257	12/12	12/12	114-270	10/12	10/10	129-315

CREST	12/12	25/25	1.2	12/12	23/23	1.2	11/12	15/15	0.9	9/12	11/11	0.8	5/12	5/5	1.1

Sensitivity, specificity, and breakpoint error of interchromosomal breakpoint calls across all five coverage levels for all five programs. SE = sensitivity (true events captured/ # true events), SP = specificity (# predictions that capture true events/ # predictions), ABE = average breakpoint error. Since SVDetect and GASV predict a range of breakpoints, we reported their average breakpoint errors as ranges. BreakDancer and CREST tended to have redundant predictions, but we did not count this against specificity since redundant predictions typically supported a single common event. These redundancies could easily be merged into a single prediction. The CREST package includes a program to remove redundancies, but BreakDancer does not address these redundant predictions.

**Table 4:** Simulated dataset 2 results (75 bp reads)

	40X			30X			20X			10X			4X		
**Method**	**SE**	**SP**	**ABE**	**SE**	**SP**	**ABE**	**SE**	**SP**	**ABE**	**SE**	**SP**	**ABE**	**SE**	**SP**	**ABE**

B-phon	9/9	9/9	3.5	9/9	9/9	3.4	8/9	8/8	1.6	9/9	9/9	0.6	4/9	4/4	0.5

SVD	8/9	8/9	87-451	8/9	8/9	72-436	8/9	8/9	84-422	8/9	8/9	86-416	6/9	6/6	88-344

BD	9/9	14/14	169.7	9/9	14/14	170.8	9/9	13/13	155.5	7/9	10/10	129.7	1/9	1/1	199.2

GASV	9/9	9/9	73-177	9/9	9/9	75-189	9/9	9/9	75-201	8/9	8/8	81-212	7/9	7/7	101-285

CREST	9/9	15/15	1.2	9/9	14/14	1.1	8/9	8/8	0.8	7/9	7/7	1.1	1/9	1/1	1.5

The paired-end methods performed well on the lower coverage datasets. However, as expected, they are unable to accurately call precise breakpoints. Tables 3 and 4 highlight the advantage of using a method like Bellerophon that uses both paired end and split read strategies for SV detection. Bellerophon's breakpoint estimation performance was similar to that of CREST, but it was more resilient to decreasing levels of read depth. This is likely due to CREST's dependence on several sequence reads containing the variant breakpoint, whereas Bellerophon only requires at least one such read. Because the cluster region is very small compared to the size of the genome, it is unlikely that even a **single **soft-clipped subread will remap to this region by chance.

Bellerophon correctly classified all of the interchromosomal events created in our simulated data. For the events with partner predictions (i.e. "B" and "II" events), the partner breakpoints were correctly identified in all cases. As stated beforehand, Bellerophon can predict the presence of balanced translocations and complex interchromosomal insertions. GASV and SVDetect can predict balanced translocations, but for the 100 bp simulated data, they did not identify the chr3/chr6 reciprocal exchange. This was possibly due to the 1000 bp duplication at the chr6 breakpoint. However, GASV did correctly identify the reciprocal translocation in the 75 bp dataset.

### Results on prostate cancer datasets across varying levels of coverage

The results on the prostate cancer datasets are provided in Tables 5 and 6. For the PR-0508 dataset, Bellerophon's maximum sensitivity is 6/8, but one of the true events presented no signal for SV detection, so it was undetected by all five methods. Another event was captured by discordant read pairs, but its breakpoints were not spanned by soft-clipped reads. Thus, Bellerophon and CREST did not capture this event either. Overall, Bellerophon's breakpoint accuracy was slightly less than CREST, but its sensitivity was greater in all cases. Many false positives were predicted by all the methods, which were largely due to repeating elements and sequences that were homologous to true cluster regions. From our own results, we noticed that many of our false positives involved breakpoint regions that were found in the centromere or in repeat-rich regions. They were also caused by highly polymorphic regions among non-homologous chromosomes, leading to the formation of "false" chimeric clusters. Despite this, the CREST program reported far fewer false positives than the other methods because unlike the other methods, CREST exclusively uses soft-clipped reads to make its predictions. However, for breakpoints spanned by few soft-clipped reads, CREST will not perform well. In contrast, Bellerophon is less conservative in its calls since it requires fewer such reads. Thus, its false positive predictions are higher. However, by combining the paired-read and single-read signatures, Bellerophon achieved higher sensitivity than CREST, but a lower false positive rate compared to the paired-read methods. Essentially, the combined strategy maximizes sensitivity while mitigating the loss of specificity.

**Table 5:** Results on the PR-0508 dataset

	30X			22.5X			15X			7.5X			3X		
**Method**	**SE**	**SP**	**ABE**	**SE**	**SP**	**ABE**	**SE**	**SP**	**ABE**	**SE**	**SP**	**ABE**	**SE**	**SP**	**ABE**

B-phon	6/8	6/246	1.5	6/8	6/180	4.2	6/8	6/132	6.0	4/8	4/63	3.25	1/8	1/16	4.5

SVD	7/8	7/738	7.6-293	7/8	7/544	12.4-282	7/8	7/367	27-265	4/8	4/169	35.3-247	2/8	2/49	30.3-275

BD	7/8	9/490	141.2	7/8	8/343	165.1	5/8	5/233	131	2/8	2/112	161	0/8	N/A	N/A

GASV	7/8	7/538	106-393	7/8	7/392	111-402	7/8	7/270	126-418	4/8	4/133	135-439	2/8	2/43	130-425

CREST	5/8	5/55	1.1	5/8	5/43	1.1	3/8	3/37	1.0	2/8	2/17	0.5	0/8	N/A	N/A

**Table 6:** Results on the PR-1783 dataset

	30X			22.5X			15X			7.5X			3X		
**Method**	**SE**	**SP**	**ABE**	**SE**	**SP**	**ABE**	**SE**	**SP**	**ABE**	**SE**	**SP**	**ABE**	**SE**	**SP**	**ABE**

B-phon	9/9	9/290	7.7	9/9	9/212	8.3	7/9	7/156	2.6	5/9	5/86	3.7	1/9	1/39	0

SVD	9/9	9/936	19-301	9/9	9/651	24-293	8/9	8/413	27.4-276	5/9	5/193	31.5-281	1/9	1/80	43-273

BD	9/9	12/408	171.0	8/9	8/305	180	6/9	6/210	167	2/9	2/114	137	0/9	N/A	N/A

GASV	9/9	9/450	114-278	9/9	9/331	120-285	8/9	8/226	127-303	5/9	5/107	128-291	1/9	1/29	142-331

CREST	5/9	5/60	3.2	5/9	5/49	3.2	3/9	3/38	2.2	1/9	1/15	1.5	0/9	N/A	N/A

After running the classification step on the predictions from the prostate cancer datasets, we found 40 and 14 interchromosomal insertions in the PR-1783 and the PR-0508 datasets, respectively. However, these events involved at least one breakpoint that was located in the centromeres of their respective chromosomes, so we did not regard them as high confidence predictions. The PR-0508 dataset contained two apparent balanced translocations, and both were correctly identified by our method. The PR-1783 dataset contained one apparent balanced translocation that was also correctly identified. For the two datasets used in our experiments, the original studies did not state exactly how the interchromosomal breakpoints were formed, so we did not have "ground truth" information on which to compare our classification results. This is less problematic for balanced translocations, because their existence can be inferred by observing the breakpoint data. Bellerophon is better suited to locate large interchromosomal insertions (as seen with the simulated data). Regarding insertions, the lack of high quality predictions in the real data could indicate that many true events could be smaller in size, and thus the program would be less sensitive to their presence.

As stated beforehand, the combined strategy of Bellerophon is advantageous because it combines the strengths of paired-read and split read methods. Moreover, the weaknesses of both approaches are mitigated by the simultaneous use of both signals. In particular, for predicting precise breakpoints, Bellerophon only needs one soft-clipped read on each side of a fusion boundary. The CREST method performs best when many such reads are present. Compared to the other paired-end methods, Bellerophon had better specificity and near equal sensitivity, while having superior breakpoint accuracy. Given this, Bellerophon would perform well in both low and high coverage datasets. For the cancer datasets, some of the interchromosomal breakpoints identified by our method were involved in gene fusions as specified in the original study. Thus, Bellerophon is a useful tool for discovering and characterizing gene fusions caused by interchromosomal structural variants.

## Conclusions

We have presented Bellerophon, which can be used to perform paired-end screening of translocation variants, and it can be used to find the precise boundaries that define the variants as well. Our approach seeks to address the limitations inherent in methods that only consider paired reads, such as their inability to precisely call variant breakpoints. It also addresses the limitations of SV algorithms that only consider single reads, which is problematic when very few reads span a variant breakpoint. By combining the strategies of paired-read and single-read approaches to this problem, Bellerophon provides a versatile method to locate interchromosomal variants.

Bellerophon is limited in that it requires reads of sufficient length in order to trigger breakpoint resolution. This is less of a problem with methods like CREST, since it performs DNA assembly on the clipped portions of reads. Although experiments with longer reads are becoming the norm, it may still be desirable in some settings to use datasets with shorter reads. Our future work will seek to address this issue. Also, since Bellerophon does not perform assembly of clipped sequences, the breakpoint accuracy may be slightly worse than methods like CREST that form large assembled contigs from soft-clipped reads. Furthermore, the other methods used in this study can detect a wider range of structural variants than Bellerophon. However, our lab has also produced a program called SVMiner which can identify deletions and inversions in NGS data, and this framework can be easily applied to that program to improve its breakpoint prediction ability [22]. Furthermore, we will also consider adding the ability to filter out germline variants. Lastly, we will consider extending the method to incorporate the read depth signal during the classification step.

## Competing interests

The authors declare that they have no competing interests.

## Authors' contributions

MH 1) conceived the idea, 2) implemented the method, and 3) wrote the manuscript. JL initiated the study and contributed to developing the prediction algorithm.

## Supplementary Material

Additional file 1 **Supplement 1.pdf**. Click here for file

## Declarations

The publication costs for this article were funded by NIH/DC012380 and NSF/IIS1162374.

This article has been published as part of *BMC Bioinformatics *Volume 14 Supplement 5, 2013: Proceedings of the Third Annual RECOMB Satellite Workshop on Massively Parallel Sequencing (RECOMB-seq 2013). The full contents of the supplement are available online at http://www.biomedcentral.com/bmcbioinformatics/supplements/14/S5.
