# Supplementary material for: Bellerophon: a hybrid method for detecting interchromo-somal rearrangements at base pair resolution using next-generation sequencing data
Source: BMC Bioinformatics. 2013 Apr 10;14(Suppl 5):S6. doi: 10.1186/1471-2105-14-S5-S6 (PMC3622635; doi:10.1186/1471-2105-14-S5-S6)
Supplement: Additional file 1 — Supplement 1.pdf. [file 1471-2105-14-S5-S6-S1.PDF]

Bellerophon: a hybrid method for detecting interchromosomal rearrangements at base pair resolution using next-generation sequencing data

Supplementary Document

Programs, settings, and non-default parameters used in experiments

Programs: BreakDancerMax v1.1, CREST v0.0.1, SVDetect v1.3, GASV v2.0, Bellerophon v0

Measurements for each dataset

Simulated datasets (100 bp and 75 bp)

Mean mapped distance: 400 bp

Standard deviation: 80

Read length: 100 bp and 75 bp respectively

PR-0508 (observed measurements)

Mean mapped distance: 355 bp

Standard deviation: 90

Read length: 101

PR-1783 (observed measurements)

Mean mapped distance: 397 bp

Standard deviation: 53

Read length: 101

Common settings

For GASV, Bellerophon, SVDetect, and BreakDancer, predicted variants must be supported by at least 3 discordant read pairs. For CREST, we required at least 3 soft-clipped reads from each side of a variant breakpoint. All programs (except CREST) used a cutoff value of 4 units of standard deviation for filtering read pairs.[1] For Bellerophon, this is the *k* parameter. Bellerophon, GASV, and BreakDancer used alternative mapping quality score filtering of $Q \geq 30$. For SVDetect, pairs with alternative quality $Q < 30$ were removed beforehand. For CREST, we required 90% sequence similarity of soft-clipped reads to the reference (which is default). For the CAP3 assembler (used by CREST), all default settings were used [1]. Parameters relevant to dataset characteristics (e.g. read lengths, max mapped distance, min mapped distance) were set according to the measurements previously described.

Bellerophon

To trigger the breakpoint detection procedure, Bellerophon required at least 1 hit from a soft-clipped subread from either side of a variant breakpoint, and at most 5 from each side (e.g. the left side can have at most 5 hits and the right side can have at most 5 hits). For BLAT, the minimum score setting was set to 20 since Bellerophon will not attempt to remap subreads that are shorter than 20 bp. All other BLAT settings are default.

BreakDancerMax

The preprocessing and prediction steps used parameters consistent with those described under *Common settings.* All other settings are default.

GASV

The preprocessing and prediction steps used parameters consistent with those described under *Common settings.* All other settings are default.

SVDetect

Window sizes of 1000 bp were used for all datasets. The step size was 250 bp for all datasets.

CREST

For the 100 bp simulated data, PR-0508, and PR-1738, we set the minimum length for a soft-clipped read (min_sclip_len) to 20, which is default. For the 75 bp simulated dataset, we set this parameter to 10 because of the shorter read lengths.

---

[1] Standard deviation of mapped distances between mates

Classification algorithm: definitions

After the clustering and prediction phases of Bellerophon, we have a set of records that denote chimeric boundaries detected by the program.

Let *opposite*('-') return '+'
Let *opposite*('+') return '-'

Let a chimeric record *R* equal the following:
(*chr_i*, *chr_i_breakpoint*, *chr_i_strand*, *chr_j*, *chr_j_breakpoint*, *chr_i_strand*)

where the chromosome, breakpoint, and strands are provided for both participating chromosomes. A record R^x is a **mirror** of record *R* if R^x = (*chr_i*, *x_chr_i_breakpoint*, *opposite*(*chr_i_strand*), *chr_j*, *x_chr_j_breakpoint*, *opposite*(*chr_j_strand*)).
Let $L = mean + k*stdev$, where *mean* is the mean mapped distance between mate pairs in the alignment results, *stdev* is the standard deviation, and *k* is a user-specified constant. Furthermore, let *p* denote a mapped read pair, and *distance*(*p*) as the mapped distance between the mates of *p*. The mapped location of *p* is the aligned position of the first base pair of the first read of *p*.

Let *chr_i_breakpoint*(*R*) return the chromosome and coordinate of the chromosome *i* side of the chimeric record *R*. Let *chr_j_breakpoint*(*R*) be similarly defined. Let *chr_i*(*R*) return the chromosome number on the chr *i* side of the candidate variant. Let *chr_i_strand*(*R*) return the chromosome *i* strand. The *chr_j*(*R*) and *chr_j_strand*(*R*) functions are similar for chromosome *j*.

For a chimeric record R^s, let *similar*(R^s, *R*) equal **true** if all of the following conditions are true (and **false** otherwise).

1) *chr_i*(R^s) = *chr_i*(*R*)
2) *chr_j*(R^s) = *chr_j*(*R*)
3) *chr_i_strand*(R^s) = *chr_i_strand*(*R*)
4) *chr_j_strand*(R^s) = *chr_j_strand*(*R*)

Let *similar_all*(*R*) return all records that are similar to *R*. This set also includes the record *R* itself.

The classification algorithm is depicted in Figures S2 and S3.

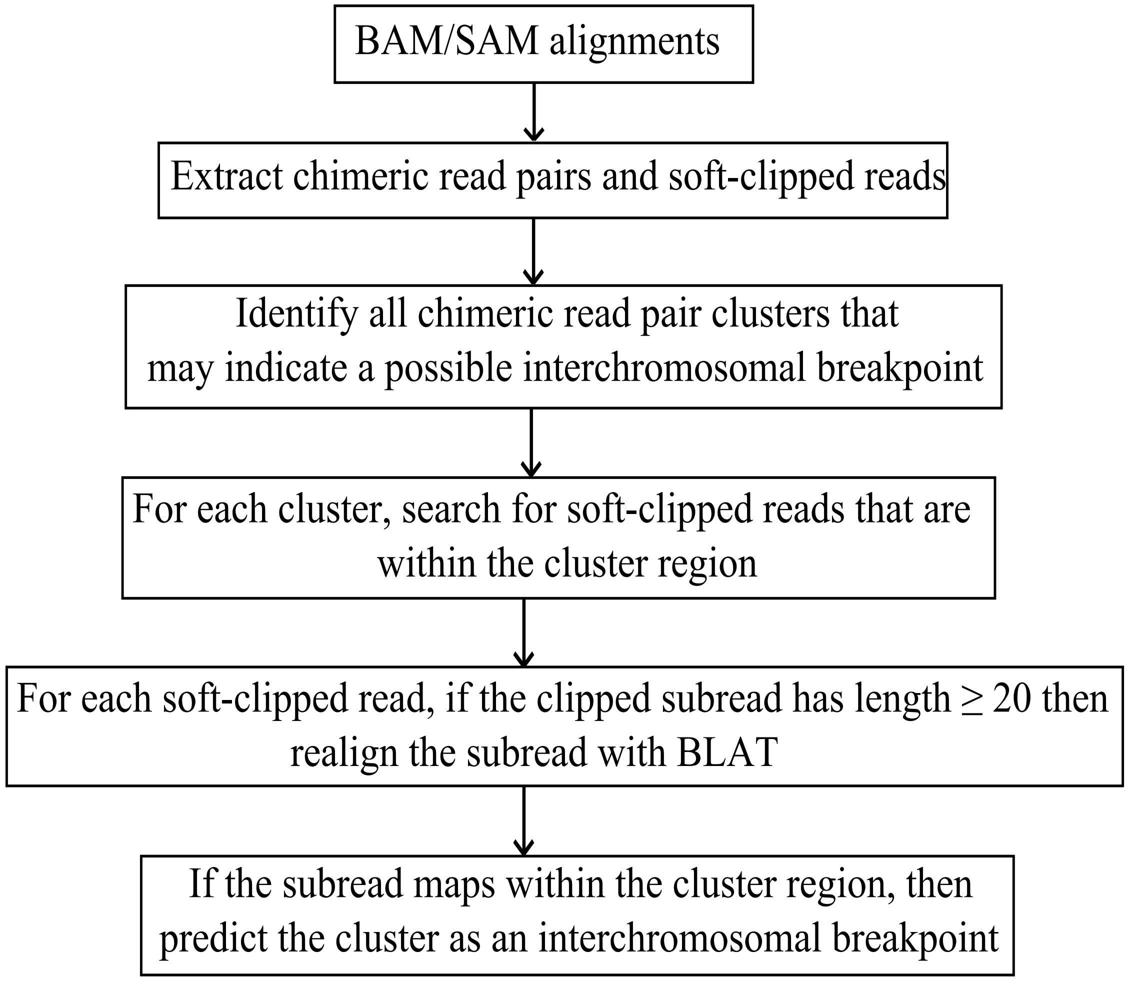

Figure S1: Flowchart for the prediction phase of Bellerophon. After this phase, the program proceeds to the interchromosomal insertion classification step described in Figure S2.

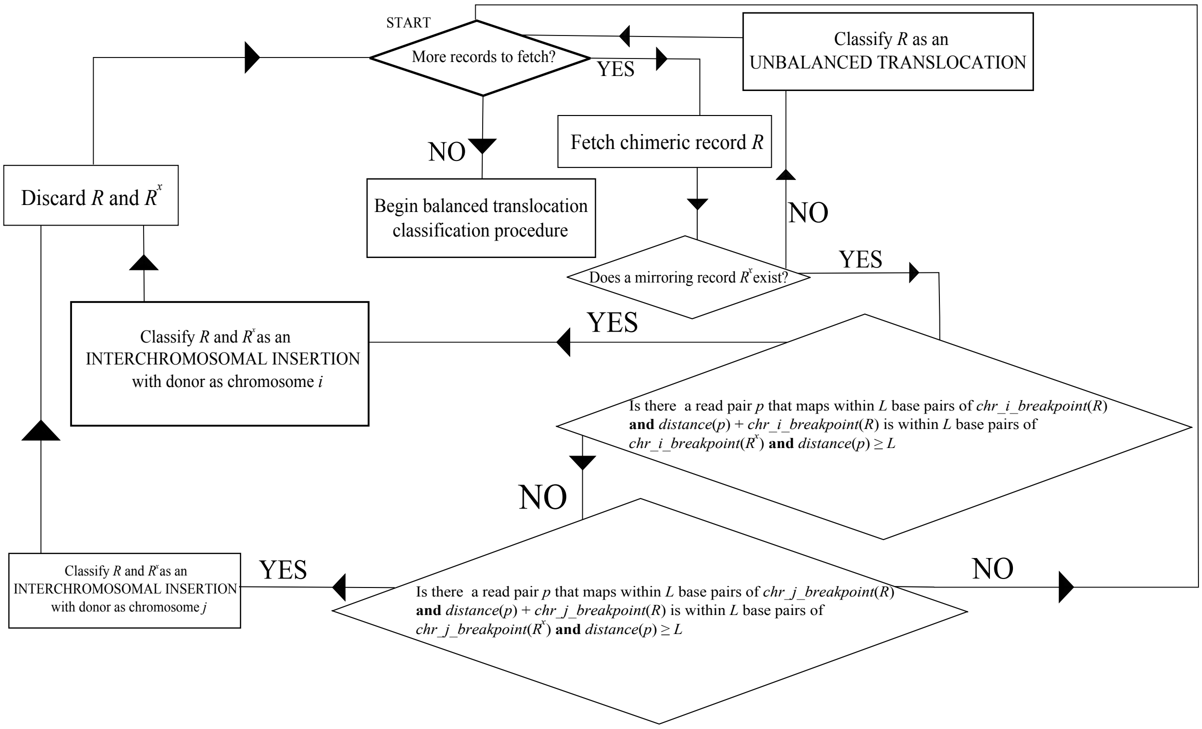

Figure S2: Flowchart for the interchromosomal insertion prediction algorithm. For mirroring records, Bellerophon first tries to classify them as interchromosomal insertions. If it cannot, it proceeds to the balanced translocation classification step depicted in Figure S3.

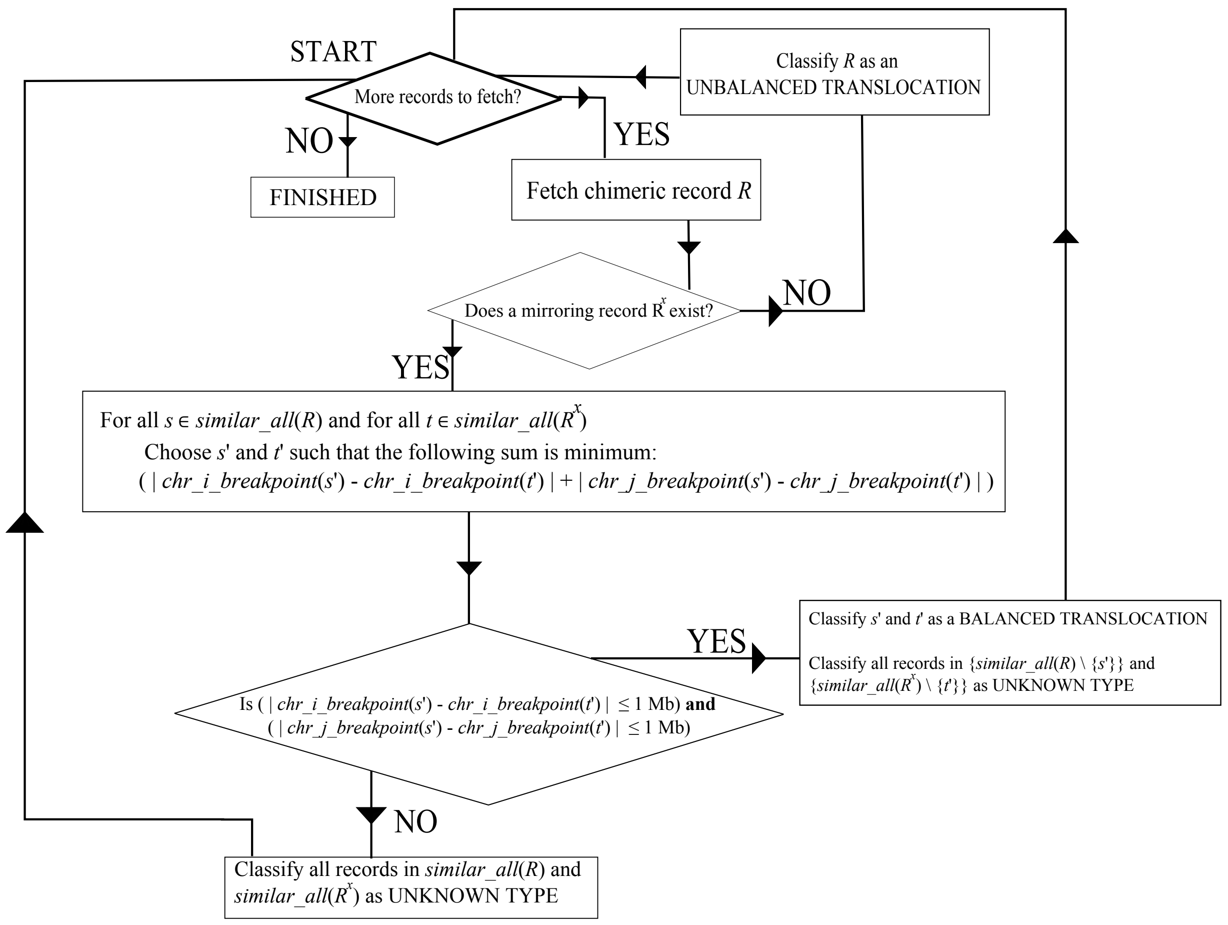

Figure S3: Flowchart for the balanced translocation prediction algorithm. Bellerophon allows for deletions and insertions of at most 1 Mb at the breakpoints.
Bellerophon will pair two mirroring records whose breakpoints are closest.
Although not depicted in the figure, Bellerophon allows for a maximum of two balanced translocation predictions between a pair of chromosomes. This allows our method to predict reciprocal translocations between two chromosomes that occur on both haplotypes.

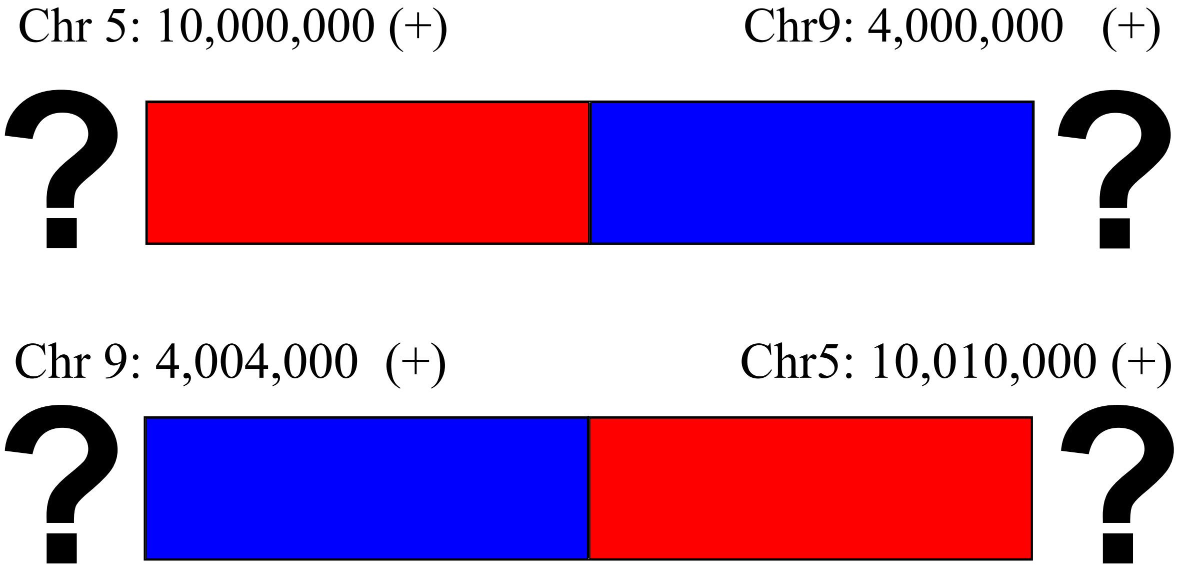

Figure S4: The uncertainty of chimeric breakpoint prediction. Many SV methods only attempt to find chimeric boundaries as shown above, but they do not attempt to classify them. The above event could denote a balanced translocation between chromosomes 5 and 9, or it could denote an interchromosomal insertion from chromosome 9 to 5.

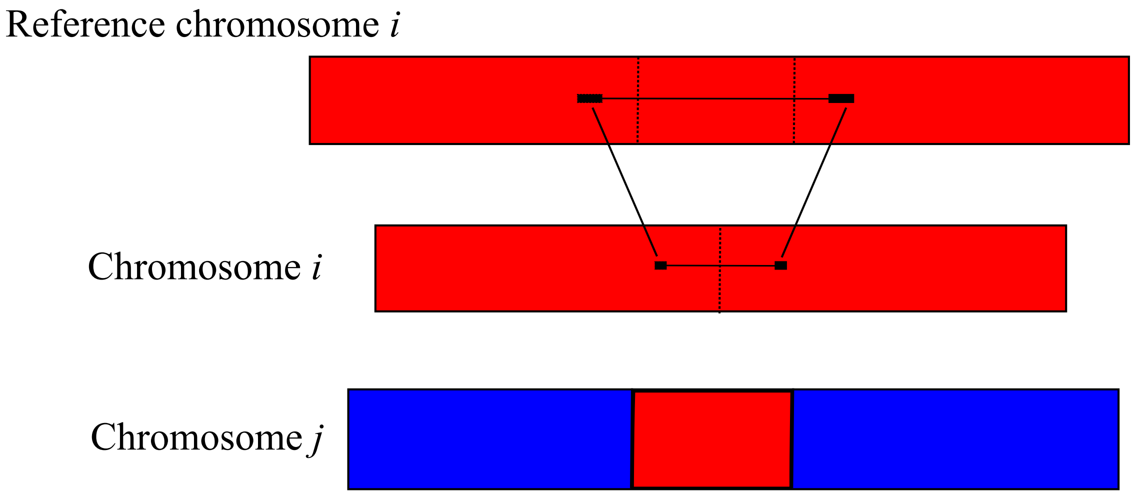

Figure S5: An interchromosomal insertion and its resulting mapping signatures. Chromosome i donated a segment to chromosome *j*. The read pair in chromosome *i* spans the donor site, and when it is mapped back to the reference, its mapped distance will be much greater than its original distance in sequencing. To infer an interchromosomal insertion, Bellerophon not only searches for mirroring records, but it searches for these long, anomalously mapped pairs (AP). If two mirroring records have no corresponding long APs, then the records could imply a balanced translocation.

References

1. Huang, X and Madan, A: **CAP3: A DNA sequence assembly program**. *Genome Res.* 1999, **9**:868-877.
